# Adolescents' Communication on Sexual and Reproductive Health Matters with Their Parents and Associated Factors among Secondary and Preparatory School Students in Ambo Town, Oromia, Ethiopia

**DOI:** 10.1155/2021/6697837

**Published:** 2021-03-16

**Authors:** Tesfaye Shibiru Bikila, Nagasa Dida, Gizachew Abdissa Bulto, Bikila Tefera Debelo, Kababa Temesgen

**Affiliations:** ^1^West Shewa Zonal Health Office, Ambo, Ethiopia; ^2^Health Education and Promotion Unit, Department of Public Health, College of Medicine and Health Sciences, Ambo University, Ambo, Ethiopia; ^3^Department of Midwifery, College of Medicine and Health Sciences, Ambo University, Ambo, Ethiopia

## Abstract

**Background:**

Sexual and reproductive health (SRH) communication is most likely to promote healthy sexual practices and to reduce risky sexual behavior among adolescents. Communication is the principal means for parents to transmit sexual values and knowledge to their children. Although there are few studies conducted on parent-adolescent communication, there is no study conducted in the town of Ambo. This study was aimed at assessing the level of parent-adolescent communication on SRH issues and its associated factors among school students in Ambo town, Oromia, Ethiopia.

**Method:**

An institution-based concurrent mixed-method cross-sectional study was conducted among 591 secondary and preparatory school students in Ambo town from February 24^th^ to March 9^th^, 2019. A systematic sampling technique was used to select the study subject. Data were collected through self-administered questionnaires, and FGD was conducted with parents of students. Data was entered using EpiData version 3.1 and exported to SPSS version 23.0 for statistical analysis. Binary and multivariable logistic regression analyses were used to ascertain the association using a 95% confidence interval (CI) and *p* value (<0.05).

**Results:**

The proportion of students who had communication on sexual and reproductive health issues with their parents was 222 (37.6%). Being female (AOR = 2.07, 95% CI: 1.40-3.07), private school (AOR = 2.77, 95% CI: 1.17-3.69), a father with secondary education (AOR = 2.93, 95% CI: 1.05-8.12) and diploma and above (AOR = 3.27, 95% CI: 1.23-8.71), considering sex education necessary (AOR = 2.83, 95% CI: 1.22-6.57), got information about SRH issues from school (AOR = 2.01, 95% CI: 1.06-2.36) and media (AOR = 2.92, 95% CI: 1.49-3.71), and mother's openness to communicate about SRH issues (AOR = 3.30, 95% CI: 1.31-4.05) were found to be significantly associated with parent-adolescent communication on SRH issues.

**Conclusions:**

The study showed that parent-adolescent communication on SRH issues is low. Being female, those from a private school, father's education, perceived importance of sex education, source of information about SRH issues (school and media), and mother's openness to communicate about SRH issues were identified to be factors associated with the communication. Therefore, the concerned body should consider the identified factors to improve the current level of parent-adolescent communication and adolescent reproductive health.

## 1. Introduction

Globally, more than 1.1 million adolescents aged 10-19 years died in 2016, over 3000 every day, mostly from preventable or treatable causes [1]. Each year, more than 1 million teenagers become pregnant and 65% of the resulting babies are born out of wedlock [2, 3]. Moreover, adolescents are more likely to engage in a wide range of high-risk sexual behaviors that can result in sexually transmitted diseases (STDs), including human immunodeficiency virus (HIV)/acquired immunodeficiency syndrome (AIDS) [4].

Sexual health is a state of physical, mental, and social wellbeing concerning sexuality across the life span that involves physical, emotional, mental, social, and spiritual dimensions [5]. Today, approximately one-fifth of the world's population is adolescents (10-19 years of age), with more than four-fifths of them residing in developing countries [6, 7]. In Ethiopia, 15.6% of them were in the age group of 10-14 and 10.6% were 15-19 years [8].

Adolescence is a time when many young people experience critical and life-defining challenges such as their first sexual experience, marriage, pregnancy, and parenthood [9]. Of all challenges, those associated with sexual maturation are the most distinctive as well as the most problematic [10]. Neglecting this population has a major implication for the future since sexual and reproductive behaviors during adolescence have far-reaching consequences for people's lives as they develop into an adult [11].

The health status of adolescents is strongly connected to several risk behaviors, which are often established during the adolescent years that end in chronic and nonchronic diseases [12, 13]. The vast majority of sexual intercourse during the adolescence period is unprotected, and therefore, the risk of unwanted pregnancy, unsafe abortion, and sexually transmitted infections (STIs) including HIV\AIDS is very high [14].

In Ethiopia, the highest prevalence of HIV infection was reported in the age group 15 to 24 (12.1%). About 60% of adolescent pregnancies in Ethiopia are unwanted or unintended pregnancies resulting from unprotected sexual intercourse and mostly end up in an unsafe abortion [15].

A study conducted in Ghana, Accra, reported that 73.6% had talked about HIV/AIDS with parents or other family members [16]. In Ethiopia, the prevalence of communication between parents and their children about sexual issues ranges from 2.6% to 36.9% [17–20].

Studies conducted in Ethiopia show that cultural factors including cultural taboos, shame, lack of communication skill, embarrassment, fear of parents, nonresponsiveness of parents, and unwelcoming nature of parents to accept young people constrained by lack of adequate knowledge, sociocultural norms, and parental belief that discussion of such issues promote premarital sexual practice were reasons that hinder communication between parent and adolescents [21, 22]. Parents' educational status, living arrangements, and level of education of respondents were also some of the factors associated with adolescent communication [19, 23].

Lack of accurate information about reproductive health and sexuality, lack of access to health services including contraception, and vulnerability to sexual abuse put adolescents at the highest risk [24]. With regard to sexuality and communication about sexual matters, perhaps now more than any other time in history, the issue of sexual health is important for virtually everyone. This is because adolescents are affected by the burden of unwanted pregnancy and its complication, HIV/AIDS, STI, and other types of sexual and reproductive ill health [25].

Sex is a subject many are uncomfortable discussing in a meaningful way, especially with children [9]. Parental communication with adolescents regarding sexuality is regarded as critical towards informing adolescents of risks and protective behaviors, providing guidelines on values and standards of behavior, and decreasing the likelihood of youths' engagement in risk behaviors [22, 26]. In Ethiopia, despite growing needs, there are inadequate sexual communication and counseling services, and researchers reported inconsistent findings.

Parents have an important role in protecting their children from risk by supervising and providing information about sexual-related risk, including the formation of attitudes and values about sexuality and the reduction of risky behaviors [27]. The finding of this study could help in improving the prevention and education program that meet the needs and concerns of adolescents. Therefore, this study was aimed at assessing the communication between parents and their children about a sexual issue in secondary and preparatory school students in Ambo town, Oromia Region, Ethiopia.

## 2. Methods

### 2.1. Study Design, Area, and Period

An institution-based concurrent mixed-method cross-sectional study was conducted among secondary and preparatory school students in Ambo town from February 24^th^ to March 9^th^, 2019. Ambo town is located in the central part of Oromia regional state, and it is the capital of the West Shoa zone. It is located 114 km to the west of Addis Ababa on the main road that leads to Wollega. The town had three urban and three rural kebeles (small administrative units). According to Ambo town administration office 2018 data, the total population of the town was 108,000 of which 53,400 were males and 54,600 were females [28].

There are 4 secondary schools, 2 (one public and one private) preparatory schools, and 21 (10 public and 11 private) elementary schools found in Ambo town. The total numbers of students attending grades 9-12 during the 2018/19 calendar year were 9,457; of these, 5,033 and 4,224 were males and females, respectively. There were 3 youth service centers in the town [28].

### 2.2. Source and Study Population

The source populations were students from grade 9 to grade 12 attending secondary and preparatory schools in Ambo town in the academic year of 2018/2019. Students from grades 9 to 12 attending secondary and preparatory schools in Ambo town and who were selected by systematic random sampling from secondary and preparatory schools found in Ambo town in the academic year of 2018/2019 were our study population. For the qualitative part, the study subjects (discussants) were purposively selected parents who have adolescent students in preparatory and high schools and were involved in the focus group discussions to explore communication on SRH issues. All students aged 10-19 years and who were married or have no parents were excluded from the study.

### 2.3. Sample Size Determination

The sample size for the quantitative part was determined using the single population proportion formula with the assumption of 95% two-sided confidence level (CI), 36.9% of the proportion of parent-adolescent communicating on sexual and reproductive health in Debre Markos Town (2014) [19] and 5% marginal error.

*n* = (*Zα*/2)^2^*P*(1 − *P*)/*d*^2^ = (1.96)^2^∗(0.369)∗(1 − 0.369)/(0.05)^2^ = 358.

By considering a 10% nonresponse rate and 1.5 design effect, the final sample size was 591.

### 2.4. Sampling Procedure

A multistage sampling technique was employed to recruit study participants. In the beginning, schools in the town were stratified into public and private ones. Then, three out of four governmental schools and one out of two private schools were randomly selected. Then, schools were further stratified by the grade of students. Then, 75% of sections from all grades were selected by simple random sampling technique using the lottery method. Furthermore, the number of students selected from each section was proportionally allocated according to the number of students in the class. Finally, a systematic random sampling technique was used to select the study unit from each selected section by using the list of students from the roster as a sampling frame. Accordingly, every 9^th^ student was selected after randomly identifying the first respondent from the list of the first 9 students on the roster, then every 9^th^ respondent was selected. For the qualitative part, FDG was conducted among purposively selected parents who had adolescent students in preparatory and high schools.

### 2.5. Variables of the Study and Measurements

Parent-adolescent communication on SRH issues was our dependent variable. Variables, such as Sociodemographic characteristics, knowledge on SRH, sexual attitude and behaviors, and sociocultural factors were our independent variables.

- *Parent-adolescent communication on sexual and reproductive health*: when students discussed SRH issues of at least two topics with their parents (about condom, STI/HIV, sexual intercourse, menarche, unwanted pregnancy, and contraceptive method) in the last 12 months [18].
- *Knowledgeable on SRH*: those students who score greater than the mean of sexual and reproductive health-related questions while those students who score the mean or below the mean were considered as not knowledgeable on SRH issues [29].

### 2.6. Data Collection Tool and Techniques

Four experienced data collectors who have completed grade 12 and fluent in the Afan Oromo language were recruited. Two nurse professionals were recruited as supervisors, and they were responsible for leading the whole situation of data collection processes. Data were collected through a self-administered questionnaire by using a structured Afan Oromo version questionnaire. The questionnaires were originally adapted by reviewing different related literature [18, 30–35] in English and translated into Afan Oromo and back to English by different experts to check for consistency. The questions consisted of sociodemographic characteristics, knowledge on SRH issues, adolescent's perception of SRH issues and their behaviors, and adolescent-parent communications on SRH issues.

For the qualitative part, four focus group discussions stratified into male groups and female groups were carried out among purposively selected parents who had adolescent students in preparatory and high schools. A semistructured interview guideline was used to lead the discussion. Two trained health workers moderated the FGD sessions. The FGD consisting of eight to ten discussant mothers and fathers were conducted separately to let them freely express their ideas which also helps to increase the quality of information that can be generated. During the data collection process, the researcher took notes in written form and recorded the voice of the respondent depending on their willingness to be recorded, and the collected data was transcribed for further analysis. The data collection process continued till all the research questions were answered and the point of saturation was reached.

### 2.7. Data Quality Control and Management

The training was given to both data collectors and supervisors for two days by investigators. The questionnaire was pretested on 5% of students at secondary and preparatory schools in Guder town, which is located 15 kilometers away from the study area, after which, necessary modifications were made. Daily supervision was conducted throughout the data collection period.

### 2.8. Data Processing and Analysis

The collected data were checked for completeness, coded and entered into EpiData version 3.1, and exported to SPSS windows version 23.0 for statistical analysis. Descriptive statistical analysis was used to compute frequency, percentage, and mean for independent and dependent variables. Binary logistic regression analysis was used to ascertain the association between explanatory variables and outcome. Variables with a *p* value of ≤0.25 in the bivariate analysis were entered into a multivariable logistic regression analysis to determine the independently associated factor of adolescent-parent communication on sexual and reproductive health issues. Variables with a *p* value less than 0.05 in the multivariate analysis were considered as significantly associated. The qualitative data were analyzed according to its thematic area and were triangulated with the quantitative results.

For qualitative data, data analysis was started alongside the data collection process through transcribing, noticing important and developing new issues that were needed to be included in the process, reviewing the collected data and memos, and cleaning data throughout the process. The data that were collected through focus group discussions were transcribed, coded, and thematically analyzed based on the procedure. After the data collection in Afan Oromo, the researchers transcribed it into English and the coding process followed by being guided by the research questions. After coding, categorization followed, and the coded data were categorized into similar and related categories. The coded data were categorized based on their similarity or relatedness. The researchers categorized the coded data depending on the similarity and relationship of codes and summarized it manually.

### 2.9. Ethical Considerations

Ethical clearance was obtained from the research and ethical review committee of the College of Medicine and Health Sciences, Ambo University, before the data collection. A support letter was also obtained from the Ambo town educational office to Ambo preparatory and secondary school administrations. Participants were told that they have the right to discontinue or refuse to participate in the study. Written assent was obtained from students who were under 18 years and consent was also obtained from their parents. Written informed consent was obtained from adolescents who were 18 to 19 years of age.

## 3. Results

### 3.1. Sociodemographic Characteristics of the Respondents

Five hundred ninety-one students participated in the study, of which five hundred ninety (590) participants completed the questionnaire fully with a response rate of 99.8%. More than half 315 (53.4%) of the respondents were female. Three-fourths, 438 (74.24%), of students were between 17 and 19 years old. The mean age of the study participants was 17.27 with a standard deviation of ±1.22 years. Almost half, 317 (53.73%), of the respondents were from grades 9 and 10. Nearly all, 566 (95.93%), of them were from the Oromo ethnic group. Furthermore, about 469 (80.17%) of adolescents' mothers were private employees. The majority, 470 (79.66%), of respondents were living with both of their parents. Four hundred forty-one (74.75%) of study participants reported that they did not receive pocket money monthly from their parents. In about 174 (30.16%) of respondents, their mothers had no formal education (Table 1).

### 3.2. Adolescent-Parent Communication on Sexual and Reproductive Health Issues

Overall, 296 (53.62%) of the respondents discussed at least two topics about SRH issues at least with the father, mother, sister, peer, teacher, and other individuals in the past 12 months, whereas 256 (46.38%) did not discuss the issue with anyone. The proportion of adolescents who had communicated with their parents regarding sexual and reproductive health issues on at least two topics in the last 12 months was 222 (37.6%). Two hundred seventy-nine (47.29%) of the respondents reported that they had discussed contraceptive methods. Out of 590 respondents, 231 (39.15%) of the students had discussed sexual intercourse. However, 160 (69.26%) of these respondents had discussed with their friends and 50 (21.65%) with their mother. Near to half of the participants, 263 (44.58%), had discussed unwanted pregnancy. One hundred seventy (64.64%) of the respondents had discussed with their friends/peers and 89 (33.84%) with their mother (Table 2).

The data generated from FGD discussants indicated that they have communicated on various components of sexual and reproductive health issues, though the communication varies among families. Some parents communicate transparently with their children. A 36-year-old male participant said, “I transparently convey my communication and advice to my sons who are 16 and 18 years old. I openly and transparently convey my message regarding SRH issues at an appropriate age, conditions, and environment. I always transparently and adequately inform and give them feedback and information. Usually, when we are around the table during the evening, I communicate with them the risks and problems associated with teenage pregnancy, early sexual engagement, peer pressure, and risky behaviors based on existing problems in our country and facts in the society. We do this to help our children to be successful in their education and future life.”

On the contrary, the data generated from mothers' FGD discussants revealed they communicate about SRH issues with adolescents; however, they do not openly and transparently communicate as needed and they only focus on few topics. A 35-year-old mother described her experience as “I have not shared with my children my experience even I have not discussed with them in detail about sexual and reproductive health issues, my discussion mostly focused on the impact of negative sexual and reproductive health outcome and changes during puberty”.

The data generated indicated that the way parents used to grow up their children influences sexual and reproductive health communication. During focus group discussions, most of the discussants agreed and noted that children should be encouraged to talk, express their ideas, and listened to starting from when they were kids. One father participant explained that “Previously when my children were kids, I was not caring, supportive, and close to them. I did not know the importance of these during that time. My children are not open and free, even to communicate with my other issues. They are not close to issues and even academic issues too. I encourage them to communicate with me openly but they don't. These resulted from the way we used to rear up them.”

### 3.3. Conditions That Hinder Parent-Adolescent Communication

Out of 263 (44.58%) respondents who had not discussed contraceptive methods, 109 (43.5%) and 89 (28.62%) reported their reason as do not know and shameful to discuss such issues with parents, respectively. Nearly half, 275 (46.69), of the respondents did not discuss sexual intercourse. The reasons they mentioned for not discussing sexual intercourse with their parents were shamefulness, 140 (38.89%), and do not know about the topic, 63 (17.50%) (Table 3).

The data generated from FGD participant parents identified (explored) the following hindering factors for parent-adolescent communication on SRH issues.

Parents stated that in the past, communication on sexual and reproductive health issues was considered culturally taboo, and adolescents were not allowed to openly talk about SRH-related issues. One female participant said that “Cultural values and norms are the most hindering conditions, I ashamed to rise in such a way that not interested to discuss the issues of sexuality topics. And these have created an influence on SRH communication I have with my children”.

The data generated from FGD with parents specified that still many people are ashamed to talk about SRH issues especially about sexual intercourse and opposite-sex relationship. Moreover, it was indicated that our cultural practice and attitudes have not allowed such communication and have an influence on SRH communication. One male participant illustrated that “Some of our cultural attitudes are negatively associated with such communication. Surprisingly, it is not common in our culture to openly talk about SRH issues, even to call or orally describe our reproductive organs by their names. Such practices of the society negatively impact discussion on SRH issues in a family context.”

Business or lack of time to communicate and spend time with adolescents was the other hindering condition identified on FGDs. Parents indicated that sometimes, they were very busy with different income-generating and household activities/workload. And these prevented them from communicating and giving adequate time to their children.

### 3.4. Source of Information on SRH Issues

**Figure 1:**
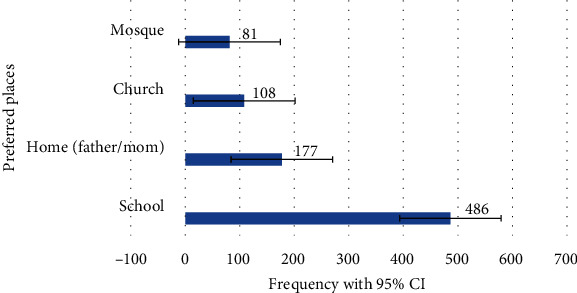
Source of information preference for sexual education and information by adolescents in Ambo Town, Oromia Region, Ethiopia, 2019.

Around two-thirds, 385(65.25%), of adolescents have gotten information about SRH from different sources. Only 28 (4.75%) reported do not know where to get the information. Regarding the source of information on reproductive health issues, 247 (59.95%) of respondents get the information from school, 282(68.45%) from media (television, radio, and magazine), 72 (17.48%) from family, and 165 (40.05%) from their peers. The majority of adolescents, 486 (82.37%), prefer school as a source of information on SRH issues (Figure 1).

### 3.5. Knowledge of Respondents on SRH Issues and Sexual Behaviors

The majority of respondents, 489 (82.88%), knew about STI/HIV/AIDS. HIV/AIDS was the most commonly known STI, 423 (86.50%), followed by syphilis, 262 (53.58%). Nearly two-thirds, 388(65.76%), of students knew about at least one contraceptive method (Table 4). According to data obtained from parents' FGD, the information that they get from religious organizations in the form of training/teaching helped them communicate with adolescents on SRH issues. One father FGD participant described that “I had communicated with my adolescents when I got education or training from church on SRH issues. Our church taught me about HIV/AIDS, relationships, risky behaviors, and activities related to SRH. When I got such kind of opportunities, it paves the way for me to start a discussion and to share what I get for my children.”

One hundred ninety-six (33.22%) of the students believed that it is normal and acceptable to have sexual feelings during the adolescent period. Regarding their practice, 76 (12.88%) of the students had a history of sexual intercourse, of which 30 (39.48%) had more than one sexual partner. Of those who had a history of sexual intercourse, 49 (64.47%) of them made sex without a condom.

Three hundred eighty-eight (65.76%) of students strongly agreed sex education is necessary (Table 4). The data generated from FGD discussant parents agree on the constructive view of parents on the communication of SRH issues is important for the communication to take place. None of the discussants accepted premarital sex. One female participant said, “Attitude of parents and adolescents on the importance of parent-adolescent communication on SRH issue was facilitating condition for their communication. Giving worth for communicating SRH issues and having the interest to communicate facilitates SRH communication. I agreed on girls maintained their virginity. It is not acceptable according to our culture and religion to have premarital sex and daughter should keep their virginity until marriage.”

### 3.6. Factors Associated with Parent-Adolescent Communication on SRH Issues

In bivariate analysis, variables such as age group, sex, father's educational status, type of school, father's occupation, knowledge of age at which menses starts, knowledge of STDs, accepting the necessity of sex education, source of information about SRH (school, media, and home), parent communication skills on SRH issues, and mother's openness to communicate about SRH issues were found to be significantly associated with parent-adolescent communication on SRH issues (Table 5).

The result of the multivariable logistic regression model revealed that sex, school type, father's educational status, accepting the necessity of sex education, source of information about SRH issues (school and media), and mother's openness to communicate about SRH issues were found to be significantly associated with discussing SRH issues.

The sex of the respondents is found to be affecting their discussion on SRH issues. The odds of discussing SRH issues with parents were twice more likely in female students than males (AOR = 2.08, 95% CI: 1.405-3.07). Compared to students learning in government schools, private school students were 2.78 times more likely to have parent-adolescent SRH communication with their parents (AOR = 2.77, 95% CI: 1.17-3.69). Students with fathers having secondary education and college diploma and above were two and three times more likely to communicate with their parents on SRH issues than students with their father who could not read and write (AOR 2.93 (95% CI: 1.05-8.12) and AOR 3.27 (95% CI: 1.23-87), respectively). Adolescents, who had agreed on the importance (necessity) of sex education, were 2.83 times more likely to discuss SRH issues than those who did not agree (AOR = 2.83, 95% CI: 1.22-6.57).

Those students who got SRH information from schools were 2 times more likely to communicate on SRH issues with their parents than those who never got SRH information (OR = 2.01; 95% CI: 1.06-2.36). Similarly, students who got SRH information from mass media were almost 3 times more likely to communicate on SRH issues with their parents than those who did not get SRH information (OR = 2.92; 95% CI: 1.49-3.71). This study also revealed that those adolescents who perceived their mothers were open to communicate on SRH issues were 3.3 times more likely to communicate on SRH issues with their parents than those whose mothers were not open to communicating on SRH issues (AOR = 3.30; 95% CI: 1.31-4.05) (Table 5).

## 4. Discussion

This study assessed parent-adolescent communications on sexual and reproductive health issues and associated factors among preparatory and high school students in Ambo town, Oromia region, Ethiopia. The current study found that 37.6% of respondents had discussed at least two SRH issues with their parents in the last 12 months. This finding was in line with studies conducted in Debre Markos (36.9%) [19], Addis Ababa, Ayer Tena high school [21], and Dire Dawa town (37%) [34] but higher than a study done in Mizan (28.9%) [17] and less than a study done at Alamata, northern Ethiopia (68.2%) [36], of which the respondents had discussed at least two SRH issue with their parents.

These differences may be due to demographic and sociocultural differences and a difference in accessing SRH information. There is also a time-period gap among studies.

The findings from the qualitative data of the present study substantiate the quantitative results in which the majority of the FGD participants mentioned that they did not openly discuss SRH-related issues. Parents simply pick words or subjects related to sexuality and human reproductive health causally from mass media (television and radio) and then attempted to chat about these matters and also provide vague warnings rather than direct open discussions. FGD discussants also raised that parents are not discussing SRH issues with their adolescent children; instead, they attempted to probe into their life/experiences regarding sexuality and reproductive health which are referred to by adolescents as queries.

Concerning factors associated with parent-adolescent communication, this study has shown that female students were more likely to discuss SRH issues compared to male students. This finding is similar to the study conducted in Addis Ababa, Ayer Tena high school, and Dire Dawa town in eastern Ethiopia [21, 34]. This may be happening due to fear of risks on the side of parents that their female children are more susceptible to various problems emanating from issues related to SRHs and sociocultural. On the contrary, a study done in Benishangul Gumuz region, Bullen Woreda, Ethiopia, shows that female students were less likely to discuss SRH issues compared to their counterpart [18]. This could be due to the sociocultural difference of parents on the discussion of sexual and reproductive health issues, and female students are shier than male students to express their feeling in rural areas.

This study is also consistent with the qualitative findings. FGD discussant parents agreed that female students should discuss SRH matters more than males, since females are more susceptible to reproductive health problems than males. Parents believe/agreed that girls should maintain their virginity, and it is not acceptable according to their culture and religion to have premarital sex and the daughter should keep her virginity until marriage.

Government (public) school students were less likely to communicate on SRH issues compared to students learning in private schools. This may be due to socioeconomic differences, the educational background of parents, and the accessing of SRH information of the parents which may influence communication between parent and adolescents.

Adolescents, whose fathers attended secondary school [9–12], were more likely to communicate on SRH issues with their parents than those students whose fathers could not read and write. This is consistent with a study done in Alamata, Gurage, and Ayer Tena; secondary and preparatory student fathers with an educational status of secondary were more likely to have parent-adolescent communication on sexual and reproductive health issues compared to those who cannot read and write [21, 36, 37]. This is also true in a study conducted in Nigeria secondary school students.

Adolescents, who perceived sex education as important (necessary), were more likely to discuss SRH issues than those who did not. This could be due to knowledge of SRH issues and the perceived importance that sex education and reproductive health may matter. Those students who had gotten SRH information from schools were more likely to communicate on SRH issues with their parents than those who did not get SRH information. Similarly, students who had gotten SRH information from media were more likely to communicate on SRH issues with their parents than those who never got SRH information from media. This study was in line with a study conducted in Debra Markos town, Bullen district, and Woldia town, southern Ethiopia [18, 19, 32]. This may be due to those adolescents who had SRH information being more aware and keen to discuss SRH issues, and the information they got may give the way for initiation of communication [18, 19].

The findings from the qualitative data also support this finding. According to data obtained from children's parent's FGD, the information that they get from religious organizations and mass media in different forms helped/paved the way for them to communicate with their adolescents on SRH issues. This shows that parents who had gotten SRH information communicated more with their adolescents.

This study also revealed that those adolescents whose mothers had been open to communicating on SRH issues were more likely to communicate on SRH issues with their parents than those whose mothers were not open to communicating on SRH issues. This may be due to the difference in knowledge; most of the mothers were primary caregivers and did not feel shy when discussing sexual matters.

The implications of this study for different concerned stakeholders were though age-appropriate comprehensive sexuality education is recommended to begin in early childhood and continue through a person's lifespan, the adolescent-parent communication on SRH issues is low. The study also highlighted that there is a variation with gender, school type, father's education status, perceived importance of sex education, source of information, and mother's openness to communicate about SRH issues. Therefore, the finding calls for initiating comprehensive family life education for the adolescents and parents using information education communication and behavioral change communication materials and through mass media both at schools and in the community, promoting parent-adolescent communication on sexuality and promoting school-based clubs to enhance communication on SRH matters.

### 4.1. Limitation

Since respondents were asked about communication on SRH issues in the last twelve months, it is not free from recall bias, as they might not remember what they had discussed in the last twelve months. Since the study touches on sensitive and intimate issues, the possibility of underestimation cannot be ruled out. Finally, this study is based on cross-sectional data, which implies that the direction of causal relationships cannot always be determined.

## 5. Conclusions

This study finding showed that parent-adolescent communication on sexual and reproductive health issues in the study area is low. Adolescents discussed sexual matters more with peers than with a parent. Being female, adolescents from a private school, educational status of fathers, perceived importance of sex education, source of information about SRH issues (school and media), and mother's openness to communicate about SRH issues were found to be significantly associated with communication of adolescents with their parents about sexual and reproductive health matters.

Therefore, the stakeholders should have to work on initiating comprehensive family life education for the adolescents and parents using information education communication and behavioral change communication materials and through mass media both at schools and in the community, promoting parent-adolescent communication on sexuality and promoting school-based clubs to enhance communication on SRH matters. Encouraging open discussion among family members in general and between parents and adolescents focusing on the identified predictor variables was also recommended.

## Figures and Tables

**Table 1:** Sociodemographic characteristic of adolescent students and their parents in Ambo town, Oromia region, Ethiopia, 2019.

Sociodemographic characteristics	Frequency (*n* = 590)	Percentage (%)
Sex		
Male	275	46.6
Female	315	53.4
The age group of participants		
14-16	152	25.76
17-19	438	74.24
Grade		
9-10	317	53.73
11-12	273	46.27
Type of school		
Government	387	65.59
Private	203	34.41
Religion		
Orthodox	236	40.0
Protestant	273	46.27
Wakefata	56	9.49
Others^∗^	25	4.24
Ethnicity		
Oromo	566	95.93
Amhara	20	3.39
Others^∗∗^	4	0.68
With whom do they currently live		
With both parents	388	65.76
With mother only	67	11.36
With father only	15	2.54
Friends	36	6.10
Living alone	17	2.88
Relative	67	11.36
Pocket money received monthly		
Yes	149	25.25
No	441	74.75
Marital status of a family		
Living together	470	79.66
Separated/divorced	76	12.88
Widowed	44	7.46
Father's occupation		
Private (self-employed, small scale, & farmer)	341	61.78
Government	184	33.33
Others^♣^	27	4.89
Mother's occupation		
Private (self-employed, small scale, & farmer)	471	80.51
Government	114	19.49
Father's educational level (*n* = 556)		
Has no formal education	106	17.97
Primary (grade 1 to 8)	85	14.41
Secondary (grade 9 to 12)	152	25.76
Diploma and above	247	41.86
Mother's educational level (*n* = 577)		
Had no formal education	174	29.49
Primary (grade 1 to 8)	152	25.76
Secondary (grade 9 to 12)	109	18.48
Diploma and above	155	26.27
Family size		
2-6	370	62.71
7-14	220	37.29

^∗^Catholic, Muslim; ^∗∗^Gurage and Tigre; ^♣^nongovernmental organization and daily laborer.

**Table 2:** Discussion on SRH issues among adolescents and their family and others in secondary and preparatory school students in Ambo Town, Oromia region, Ethiopia, 2019.

Types of SRH issues	Discussed	With whom SRH was discussed						
	Yes	Father^∗^	Mother^∗^	Peers^∗^	Sister^∗^	Brother^∗^	Teacher^∗^	Others^∗^
Contraceptive	279(47.29)	103(36.92)	125(44.80)	149(53.49)	81(29.03)	65(23.30)	35(12.54)	8(2.87)
STI/HIV	228(38.64)	43(18.86)	62(27.19)	149(65.35)	46(20.18)	36(15.79)	59(25.88)	8(3.51)
Sexual intercourse	231(39.15)	20(8.66)	50(21.65)	160(69.26)	30(12.99)	30(12.99)	33(14.29)	9(3.90)
Unwanted pregnancy	263(44.58)	29(11.03)	89(33.84)	170(64.64)	64(24.33)	34(12.98)	56(21.37)	14(5.34)
No sex before marriage	379(64.24)	61(16.09)	138(36.41)	260(68.60)	85(22.43)	64(16.89)	77(20.32)	17(4.49)
Condom	259(43.90)	30(11.54)	45(17.31)	167(64.23)	20(7.69)	41(15.77)	73(28.08)	13(5.00)
Puberty	366(62.03)	53(14.44)	99(26.98)	235(64.03)	64(17.44)	58(15.80)	103(28.07)	14(3.81)
Menstrual period	296(50.17)	19(6.42)	135(45.61)	183(61.82)	79(26.69)	28(9.46)	54(18.24)	9(3.04)

^∗^Multiple responses and percentage in parenthesis.

**Table 3:** Reasons reported by adolescents for not discussing SRH issues with their parents among secondary and preparatory school students in Ambo Town, Oromia region, Ethiopia, 2019.

Types of SRH issues	Not discussed	Reasons why SRH was not discussed, parents							
		Culturally unacceptable^∗^	Shame^∗^	Knowledge gap^∗^	Communication skill gap^∗^	Not listen^∗^	Difficult & embarrassing^∗^	Do not know^∗^	Others^∗^
Contraceptive	263(44.6)	48(15.4)	89(28.6)	54(17.4)	34(10.9)	31(9.9)	31(9.9)	109(35.1)	15(4.8)
STI/HIV	279(47.3)	38(10.5)	85(23.5)	66(18.2)	29(8.0)	19(5.3)	30(8.3)	135(41.9)	8(2.2)
Sexual intercourse	315(53.4)	63(17.5)	140(38.9)	52(14.4)	20(5.6)	16(4.4)	45(12.5)	119(33.1)	6(1.7)
Unwanted pregnancy	275(46.7)	57(17.5)	89(27.3)	56(17.2)	16(4.9)	14(4.3)	25(7.7)	135(40.2)	9(2.8)
No sex before marriage	168(28.5)	30(14.2)	62(29.4)	39(18.5)	12(5.7)	9(4.3)	23(10.9)	83(39.3)	5(2.4)
Condom	285(48.3)	49(14.8)	96(29.0)	52(15.7)	23(6.9)	18(5.4)	42(12.7)	127(38.4)	5(1.5)
Puberty	161(27.3)	18(8.0)	47(20.9)	33(14.7)	23(10.3)	19(8.5)	26(11.6)	97(43.3)	1(0.5)
Menstrual period	255(43.3)	41(13.9)	100(34.1)	50(17.1)	27(9.2)	23(7.8)	23(7.8)	102(34.8)	9(3.1)

^∗^Multiple responses and percentage in parenthesis.

**Table 4:** Adolescent knowledge on SRH issues and sexual behaviors among secondary and preparatory school students in Ambo Town, Oromia region, Ethiopia, 2019.

Variables	Frequency (*n*)	Percentage (%)
Know about the menstrual cycle		
Yes	372	63.05
No	218	36.95
Perceived age at which menstrual starts (*n* = 372)		
11-12	54	14.52
13-14	250	67.20
15-16	68	18.28
Your feeling when first menses comes(*n* = 315)		
Tension	92	29.21
Fear	110	34.92
Pleasure	42	13.33
Being diseased	45	14.29
Shame	19	6.03
Have not seen it yet	7	2.22
Know sexually transmitted infection		
Yes	489	82.88
No	101	17.12
Types of STD participants know (*n* = 489)^∗^		
Chancroid	47	9.61
Syphilis	262	53.58
Gonorrhea	243	49.69
Lymphogranuloma venereum	34	6.95
HIV	423	86.50
Herpes simplex	42	8.59
Know any contraceptive methods		
Yes	388	65.76
No	202	34.24
Type of contraceptive they know (*n* = 388)^∗^		
Pills	262	67.53
Depo (injectable)	185	47.68
Implants	186	47.94
IUCD (loop)	150	38.66
Condoms	176	45.36
Abstinence	103	26.55
Calendar	19	4.90
Having sexual feeling during adolescence is normal		
Yes	196	33.22
No	321	54.41
I do not know	73	12.37
Have you started sexual intercourse?		
Yes	76	12.88
No	514	87.12
Age when sexual intercourse was started (*n* = 76)		
13-15	18	23.68
16-18	58	76.32
Number of sexual partners ever had (*n* = 76)		
One	46	60.53
Two and above	30	39.48
Condoms were used during intercourse		
Yes	27	35.53
No	49	64.47
Do you accept premarital sex?		
Yes	55	9.32
No	535	90.68
Do you believe sex education is necessary?		
Yes	388	65.76
No	158	26.78
I do not know	44	7.46

^∗^Multiple responses.

**Table 5:** Factors associated with parent-adolescent communication on SRH issues among secondary and preparatory school students in Ambo Town, Oromia region, Ethiopia, 2019.

Variables	Parent-adolescent SRH communication		Crude OR (95% CI)	Adjusted OR (95% CI)	*p* value
	Yes	No			
Sex					
Male	135	180	1	1	0.001^∗^
Female	87	188	1.62(1.15, 2.27)	2.03(1.37, 2.99)	
Age group					
14-16	71	81	1.66(1.14, 2.42)	1.73(0.78, 1.94)	0.356
17-19	151	287	1	1	
School type					
Government	95	88	1	1	0.009^∗^
Private	127	280	2.38(0.64, 3.40)	2.77(1.17, 3.69)	
Father's occupation					
Private^∗^	63	97	1.60(1.05, 2.43)	0.83(0.47, 1.46)	0.530
Government	88	96	2.26(1.51, 3.36)	2.44(0.66, 3.30)	0.493
Others	71	175	1	1	
Father's education					
No formal education	27	79	1	1	
Primary (grade 1 to 8)	26	59	1.28(0.56, 3.56)	1.84(0.64, 5.29)	0.256
Secondary (grade 9 to 12)	51	101	1.47(0.10, 4.48)	2.93(1.05, 8.12)	0.039^∗^
Diploma and above	118	129	2.67(0.59, 4.26)	3.27(1.23, 8.71)	0.017^∗^
Know the age at which menses starts					
Yes	153	219	1.50(0.46, 2.94)	1.28(0.77, 2.52)	0.327
No	69	149	1	1	
Know sexually transmitted diseases					
Yes	194	295	1.71(0.36, 1.9)	1.96(0.80, 2.32)	0.249
No	28	73	1	1	
The necessity of sex education					
Yes	158	230	1.48(1.12, 4.86)	2.83(1.22, 6.57)	0.015^∗^
No	64	138	1	1	
Got information about sexual matters from school					
Yes	116	133	1.98(0.93, 2.05)	2.01(1.06, 2.36)	0.023^∗^
No	106	235	1	1	
Got information about sexual matters from media					
Yes	139	143	2.63(1.27, 3.02)	2.92(1.49, 3.71)	0.001^∗^
No	83	225	1	1	
Got information about sexual matters at home					
Yes	33	39	1.47(0.41, 2.11)	2.00(0.53, 2.88)	0.986
No	189	329	1	1	
Mother open to discuss SRH issues					
Yes	62	40	3.17(1.73, 4.58)	3.30(1.31, 4.05)	0.004^∗^
No	160	328	1	1	

^∗^Significant association *p* < 0.05.

## Data Availability

The datasets used and/or analyzed during the current study are available from the corresponding author upon request.

## Abbreviations

AIDS: Acquired immunodeficiency syndrome
FGD: Focus group discussion
HIV: Human immunodeficiency virus
STI: Sexually transmitted infection
STD: Sexually transmitted diseases
SRH: Sexual and reproductive health.
